# Growth performance, nutrient digestibility, intestinal morphology, cecal mucosal cytokines and serum antioxidant responses of broiler chickens to dietary enzymatically treated yeast and coccidia challenge

**DOI:** 10.1186/s40104-023-00846-z

**Published:** 2023-04-10

**Authors:** Emmanuel Oluwabukunmi Alagbe, Hagen Schulze, Olayiwola Adeola

**Affiliations:** 1 Department of Animal Sciences, Purdue University, West Lafayette, IN 47907 USA; 2 Livalta, Peterborough, Cambridgeshire, PE2 6FL UK

**Keywords:** Broiler chickens, Coccidia, Goblet cells, Growth performance, Health, Oocyst, Peptides, Postbiotics, Yeast

## Abstract

**Background:**

There is a growing search for natural feed additives to alleviate the deleterious effects of coccidia infection in poultry production. This study aimed to investigate the effect of enzymatically treated yeast (ETY) on the growth performance, nutrient digestibility, intestinal morphology, antioxidative status, and cecal mucosa cytokines of coccidia-challenged broiler chickens.

**Methods:**

From d 1 to 14 post hatching, 480 broiler chickens were allocated to 3 corn-soybean meal-based experimental diets with increasing concentrations of ETY (0, 1, or 2 g/kg). The experiment was designed as a randomized complete block design with body weight (BW) used as a blocking factor. On d 14 post hatching, the birds were re-randomized within each of the 3 experimental diets. Each of the 3 diet groups was split into a challenge or no-challenge group. This resulted in a 3 × 2 factorial arrangement of treatments. The coccidia challenge was administered on d 15 by an oral gavage.

**Results:**

Dietary ETY improved (*P* < 0.05) the G:F of birds on d 21 regardless of the challenge state and linearly increased (*P* < 0.01) the apparent ileal digestibility of dry matter (DM), nitrogen, and gross energy (GE). The coccidia challenge decreased (*P* < 0.05) BW gain and feed intake of broiler chickens and reduced (*P* < 0.01) the total tract retention of DM, GE, and nitrogen. The coccidia challenge increased (*P* < 0.01) the mRNA gene expression of *TNFα*, *IL-1β*, *IL-10*, and *IL-6* in the cecal mucosa. There was a tendency (*P* = 0.08) for ETY to linearly reduce *IL-1β* expression. Additionally, ETY supplementation increased (*P* < 0.05) the gene expression of *OCLN*. Serum catalase increased (*P* < 0.05) with dietary ETY in broiler chickens on d 21. Dietary ETY linearly increased (*P* < 0.05) the ileal villus height to crypt depth ratio, and ileal goblet cell density in broiler chickens. The ileal and excreta oocyst counts decreased (*P* < 0.01) with increasing supplementation of dietary ETY in coccidia-challenged broiler chickens on d 21.

**Conclusions:**

Dietary ETY enhanced nutrient utilization and augmented intestinal development in broiler chickens. However, dietary ETY did not completely attenuate the adverse effects of a coccidia challenge in broiler chickens.

**Supplementary Information:**

The online version contains supplementary material available at 10.1186/s40104-023-00846-z.

## Background

Coccidiosis has a deleterious impact on poultry production all over the world. Yearly global losses attributable to coccidiosis are more than $12.4 billion [1]. This disease leads to reduction in weight, decreased feed intake, attenuation of immunity, and intestinal disruption in broiler chickens [2, 3]. The prevalent prevention and treatment approach for coccidiosis is with vaccines and drugs. However, due to the high cost of coccidia vaccines and the increasing drug resistance in *Eimeria* species, there is a need to explore innovative approaches in combating this ubiquitous poultry disease [4, 5].

Yeast (*Saccharomyces cerevisiae*) derived products such as probiotics, prebiotics or postbiotics have been identified for their health-promoting characteristics [6]. These yeast products can improve immunity, enhance intestinal health, and promote the growth performance of broiler chickens [6, 7]. Research has also shown that they could help ameliorate some of the deleterious effects of coccidia infection in birds [8]. The enzymatically treated yeast (ETY) used in this study is a postbiotic yeast product derived from non-genetically modified yeast strains and contains a high level of β-1,3/1,6-glucans, mannan-oligosaccharides, and protein. The ETY also contains other intracellular bioactive components with salutary properties [9]. Additionally, most of the proteins present in ETY are in the form of short peptides, which differentiates ETY from other commercially available yeast products.

Yeast-derived β-glucans and mannans have been shown to modulate health by promoting phagocytic activity and regulating innate immune responses, which may play a role in lessening coccidia-related decline in the performance of birds [10, 11]. These features are particularly augmented through enzymatic treatment of the yeast cell wall [12]. The role of peptides in promoting feed intake and stimulating appetite has also been reported [13]. Hence, these properties suggest ETY as a promising feed additive candidate to explore in *Eimeria*-related pathology. Therefore, this study was designed to evaluate the effect of dietary enzymatically treated yeast on broiler chicken performance, nutrient digestibility, intestinal morphology, antioxidative status, and cecal mucosa cytokines of coccidia-challenged broiler chickens. We hypothesized that the dietary supplementation of ETY protects broiler chickens against a coccidia challenge.

## Materials and methods

### Animals, diets, experimental design, and coccidia challenge

A total of 480 male broiler chickens (Cobb 500) were used for this experiment, with an initial body weight (BW) of 49.9 ± 3.95 g. The birds were individually weighed and tagged with identification numbers using a multi-PIT tag injector (UI Devices, Lake Villa, IL, USA). The birds were housed in electrically heated 0.35 m^2^ battery brooders (model SB 4 T, Alternative Design Manufacturing, Siloam Springs, AR, USA) maintained at 35, 32 and 27 °C from d 1 to 8, d 8 to 15, and d 15 to 21, respectively. Each cage was equipped with an attached trough feeder (60.96 cm × 8.25 cm × 11.43 cm) and individual light bulbs; the whole room was continuously lit by fluorescent lighting. Each cage had an adjustable drinker connected to a water source with two nipples. Birds had ad libitum access to feed and water during the experimental period. All experimental diets were formulated to meet or exceed the nutrient recommendation outlined in the nutrient specification for Cobb 500 birds. The diets were provided in mash form and consisted of a corn-soybean meal-based diet supplemented with ETY (LivaltaCell HY40; AB Agri Ltd., Peterborough, United Kingdom) at 0, 1, or 2 g/kg (Table 1). The enzymatically treated yeast is made from carefully selected non-genetically modified *Saccharomyces cerevisiae* yeast strains from the Brazilian bio-ethanol industry. The enzyme used in yeast hydrolyzation is generally recognized as safe (GRAS). The characterization of enzymatically treated yeast is outlined in Additional file 1: Table S1. Diets were free from antibiotics or coccidiostats. Titanium dioxide was added to all diets at 5 g/kg as an indigestible marker.

**Table 1:** Ingredient and nutrient composition of diets, as-fed basis

Item	Concentrations of ETY, g/kg		
	0	1	2
Ingredient, g/kg			
Corn	593.4	583.4	573.4
Soybean meal (48% CP)	310.0	310.0	310.0
Soybean oil	35.0	35.0	35.0
Ground limestone	14.0	14.0	14.0
Monocalcium phosphate	13.6	13.6	13.6
Salt	4.0	4.0	4.0
*DL*-Methionine	1.8	1.8	1.8
*L*-Lysine HCl	0.2	0.2	0.2
*L*-Threonine	0.1	0.1	0.1
Vitamin-mineral premix^a^	3.0	3.0	3.0
ETY premix^b^	0.0	10.0	20.0
Titanium dioxide premix^c^	25.0	25.0	25.0
Total	1000.0	1000.0	1000.0
Calculated nutrient, g/kg			
ME, MJ/kg	13.3	13.3	13.3
CP	203.8	203.8	203.8
Ca	8.5	8.5	8.5
P	6.5	6.5	6.5
Non-phytate P	4.0	4.0	4.0
Amino acids (SID)			
Arginine	11.9	11.9	11.9
Histidine	4.7	4.7	4.7
Isoleucine	7.5	7.5	7.5
Leucine	15.5	15.5	15.5
Lysine	9.3	9.3	9.3
Methionine	4.5	4.5	4.5
Phenylalanine	8.7	8.7	8.7
Threonine	6.7	6.7	6.7
Tryptophan	2.2	2.2	2.2
Valine	8.1	8.1	8.1
Methionine + cysteine	7.1	7.1	7.1
Analyzed nutrient			
Gross energy, MJ/kg	16.8	16.8	16.9
CP, g/kg	202.2	197.6	201.6

*ETY* Enzymatically treated yeast, *ME* Metabolizable energy, *CP* Crude protein, *Ca* Calcium, *P* Phosphorus, *SID* Standardized ileal digestibility

^a^Provided the following quantities per kg of complete diet: vitamin A, 5484 IU; vitamin D_3_, 2643 ICU; vitamin E, 11 IU; menadione sodium bisulfite, 4.38 mg; riboflavin, 5.49 mg; *D*-pantothenic acid, 11 mg; niacin, 44.1 mg; choline chloride, 771 mg; vitamin B_12_, 13.2 μg; biotin, 55.2 μg; thiamine mononitrate, 2.2 mg; folic acid, 990 μg; pyridoxine hydrochloride, 3.3 mg; I, 1.11 mg; Mn, 66.06 mg; Cu, 4.44 mg; Fe, 44.1 mg; Zn, 44.1 mg; Se, 300 μg

^b^One gram ETY added to 9 g of corn to make 10 g ETY premix. 10 g/kg of ETY premix delivered 1 g/kg of ETY; 20 g/kg of ETY premix delivered 2 g/kg of ETY

^c^Prepared as 5 g TiO_2_ plus 20 g corn

**Fig. 1:**
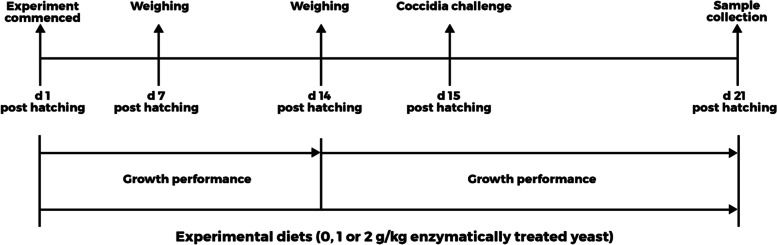
Diagram depicting the experimental procedure. Broiler chickens were fed experimental diets supplemented with increasing concentrations of ETY from d 1 to 14 post hatching. On d 14, the birds were re-randomized and separated into a challenge and no-challenge group. On d 15 post hatching, birds in the challenge and no-challenge groups were orally gavaged with 1 mL of *Eimeria* oocysts and 1% PBS, respectively. Growth performance was measured from d 1 to 14 and d 14 to 21 post hatching. Excreta, blood, tissue, and ileal digesta samples were collected on d 21 post hatching

From d 1 to 14 post hatching, the birds were assigned to 3 diets in a randomized complete block design. Body weight was used as a blocking factor. Each diet consisted of 16 replicate cages and 10 birds per cage for a total of 160 birds per diet. On d 14 post hatching, chickens were individually weighed, and leftover feed was weighed to estimate feed intake (FI). All birds within each of the 0, 1 or 2 ETY g/kg diets were combined and re-randomized (Fig. 1). With this re-randomization, the number of birds was reduced to 8 birds per cage and each of the 3 diet groups was split into a challenge or no-challenge group resulting in 6 experimental treatments. On d 15 post hatching, the birds in the challenge group were orally gavaged with 1 mL solution containing 25,000, 25,000, and 125,000 oocysts of *E. maxima*, *E. tenella,* and *E. acervulina*, respectively. The *Eimeria* oocysts were provided by Dr. Alberta Fuller from the Department of Poultry Science, University of Georgia. The birds in the no-challenge group were orally gavaged with 1 mL of 1% phosphate buffered saline (PBS; VWR International, Radnor, PA, USA). The BW ratio (the average weight of all birds from one dietary treatment to another) before the challenge was maintained across all treatments during reallotment. This resulted in a 3 × 2 factorial arrangement of treatments with 3 experimental diets (0, 1 or 2 ETY g/kg) and 2 coccidia challenge (CC) states (challenged or no-challenge) comprising 8 replicate cages and 8 birds per cage. Birds in the challenge group were separated from those in the no-challenge group by partitioning the sections with heavy-duty black contractor nylon sheets (Rural King, Lafayette, IN, USA). This was to prevent cross-contamination of litter with *Eimeria.* Additionally, entry and exit from each section were strictly controlled and followed a systematic process – from the no-challenge group to the challenge group and to exit from the facility.

### Sample collection and processing

Individual BW of birds was recorded at d 7, 14, and 21 post hatching to calculate the BW gain, FI, and gain to feed ratio (G:F). All collections also followed a systematic process (from the no-challenge group to the challenge group and exit from the facility) to prevent sample contamination with *Eimeria*. Excreta samples were collected on d 14 and 21 from pans lined with paper and placed under each cage. Excreta samples were stored at −20 °C until further analysis for eventual determination of the total tract retention (TTR) of nutrients. Fresh excreta samples were also collected from all cages into labeled 15-mL tubes (VWR International, Radnor, PA, USA) on d 21 for oocyst counting. Excreta samples were mixed with a wooden spatula and stored at 4 °C. Birds were euthanized on d 21 by CO_2_ asphyxiation. The two heaviest birds in each cage were separated for intestinal morphology and blood collection. The remaining six out of the eight birds per cage were dissected to collect ileal digesta. The distal two-thirds of the dissected ileum was flushed with distilled water into pre-labeled plastic containers. The samples were stored at −20 °C for the determination of the apparent ileal digestibility (AID) of nutrients. Portions of the flushed ileal digesta from birds were separated into labeled 15-mL tubes for oocyst counting. Samples were mixed and stored at 4 °C. Jejunal digesta were also collected from six of eight birds per cage by flushing contents from the end of the duodenum to the Meckel’s diverticulum with water into clean, pre-labeled plastic containers, and stored at −20 °C for crude mucin analysis.

Intact jejunal and ileal tissues were collected from the second-heaviest bird in each cage (10 cm of the mid jejunum or mid ileum). The excised intestinal segments were flushed with ice-cold 1% PBS, stapled to cut-out cardboard, and placed in 10% buffered formalin (VWR International, Radnor, PA, USA) for 48 h. Subsequently, the tissue sections were transferred to 50-mL tubes containing 70% ethanol and stored at 4 °C until the samples were processed. Cecal segments were excised from the second-heaviest bird in each cage, and the contents were flushed with cold PBS. They were then cut longitudinally in half to expose the lumen. Mucosa was scraped using glass slides, subsequently placed in 1.5 mL of Trizol reagent (Invitrogen, Waltham, MA, USA), and rapidly frozen in liquid nitrogen and subsequently stored at −80 °C for PCR analysis.

Furthermore, blood samples were collected from the right-wing vein of the heaviest bird per cage into non-heparinized tubes. The collected blood samples were centrifuged at 3000 × *g* for 15 min at 4 °C after clotting. Serum was aspirated and stored appropriately at −80 °C until further analyses. For lesion scoring, the entire length of the intestinal tissue sections from the heaviest bird per cage was set aside on clean laboratory cutting boards (Fisher Scientific, Waltham, MA, USA).

### Intestinal morphology

Purdue Histology and Phenotyping Laboratory (Purdue University, West Lafayette, IN, USA) processed and stained the jejunal and ileal tissue sections with Alcian blue and periodic acid-Schiff (AB-PAS). Villus height and crypt depth were measured using a microscope with an electronic camera (National Optical and Scientific Instruments, Inc., Schertz, TX, USA) and an ImageJ macro (ImageJ open-source software version 1.8). Villus height and crypt depth were measured from 5 villi per bird, and only intact and unbroken villi were considered. Villus height was defined as the distance from the tip of the villus to the crypt mouth, whereas crypt depth was defined as the distance from the base of the villus to the muscularis mucosa. Villus height to crypt depth (VH:CD) ratio was also calculated. Goblet cells (GC) were counted from the same 5 villi per bird, and the average was calculated. Goblet cell density was defined as the GC count per bird divided by the average villus height.

### Lesion scoring and oocyst counting

Intestinal tissue sections collected from one bird per cage in the challenge and no-challenge groups were evaluated for *Eimeria* lesions. The intestinal tissue regions were scored using the methodology described by Johnson and Reid [14], where 0 was considered as normal, and 1, 2, 3, or 4 signified increasing severity of coccidia infection.

Approximately 2 g of excreta or ileal digesta samples were added to 28 mL of magnesium sulfate (MgSO_4_) solution, which served as the flotation solution. The MgSO_4_ solution was prepared by adding 125 g of MgSO_4_ salt to 500 mL double distilled water. The sample in the flotation solution was homogenized using a wooden spatula and allowed to sit for 5 min. The mixture was then sieved twice using a fine-mesh kitchen sieve. Disposable plastic droppers (Fisher Scientific, Waltham, MA, USA) were used to carefully fill the chambers of a McMaster slide (Jorgensen Laboratories, Loveland, CO, USA). The slide was set on a flat table for 5 min to permit oocysts to float to the surface. Afterwards, the oocysts were counted under a microscope with an electronic camera. The results obtained were multiplied by a factor of 50 and expressed as the number of oocysts per gram of excreta/ileal digesta.

### Total RNA extraction and reverse transcription

Total RNA was extracted from the mucosa stored in the Trizol reagent following the manufacturer’s protocol. The RNA concentrations were determined using the NanoDrop 1000 (ThermoFisher Scientific, Waltham, MA, USA), and the RNA integrity was verified using 1% agarose gel electrophoresis. Subsequently, 2 mg of total RNA from each sample was reverse transcribed into cDNA using the MMLV reverse transcription reagent (Promega, Madison, WI, USA). The cDNA was then diluted 1:10 with nuclease-free water (Ambion, Austin, TX, USA) and stored at −80 °C, pending further analyses [15].

### Quantitative real-time PCR analysis

Real-time PCR of Interleukin 1β (*IL-1β*), Interleukin 6 (*IL-6*), Interleukin 10 (*IL-10*), Claudin 1 (*CLDN1*), Occludin (*OCLN*), and Tumor necrosis factor alpha (*TNFα*) genes was conducted using the Bio-Rad CFX thermocycler (Bio-Rad, Temecula, CA, USA) with the SYBR real-time PCR mix (Biotool, Houston, TX, USA) in a total reaction volume of 20 μL. The PCR reactions were incubated for 3 min at 95 °C, following which samples were subjected to 40 cycles of an amplification protocol as follows: 95 °C for 10 s, primer-specific annealing temperature for 30 s, and 95 °C for 10 s. A melt curve analysis was performed for each gene after the PCR run. The annealing temperatures and primer sequences used are listed in Table 2. Samples were analyzed in duplicates, and the acceptable coefficient of variation was set at ≤ 5%. Relative gene expression was calculated using the 2^−^^ΔΔCt^ method [16], with normalization against the housekeeping gene, glyceraldehyde-3-phosphate dehydrogenase (*GAPDH*).

**Table 2:** Sequences of primers used for the real-time PCR analysis

Target gene	Primer sequence (5′→3′)	Annealing temperature, °C	GenBank accession number	References
*IL-1β*	F: GCATCAAGGGCTACAAGCTC	57.7	NM_204524	Adedokun et al. [17]
	R: CAGGCGGTAGAAGATGAAGC			
*IL-6*	F: GAACGTCGAGTCTCTGTGCTAC	61.8	NM_204628	Current study
	R: CACCATCTGCCGGATCGT			
*IL-10*	F: GGACTATTTTCAATCCAGGGACG	53.4	NM_001004414.2	Daneshmand et al. [18]
	R: GGGCAGGACCTCATCTGTGTAG			
*CLDN1*	F: CAGACTCTAGGTTTTGCCTT	52.0	NM_001013611.2	Goo et al. [19]
	R: AATCTTTCCAGTGGCGATAC			
*OCLN*	F: TCGTGCTGTGCATCGCCATC	53.4	NM_205128.1	Goo et al. [19]
	R: CGCTGGTTCACCCCTCCGTA			
*TNFα*	F: AGATGGGAAGGGAATGAACC	55.7	AY765397	Adedokun et al. [17]
	R: ACTGGGCGGTCATAGAACAG			
*GAPDH*	F: ATGACCACTGTCCATGCCATCA	59.0	NM_204305.1	Adedokun et al. [17]
	R: AGGGATGACTTTCCCTACAGCCTT			

*F* Forward primer, *R* Reverse primer, *IL* Interleukin, *CLDN1* Claudin 1, *OCLN* Occludin, *TNFα* Tumor necrosis factor alpha, *GAPDH* Glyceraldehyde-3-phosphate dehydrogenase

### Serum and crude mucin analysis

The activities of serum glutathione peroxidase (GPX) (Lot No. ab102530, Abcam, Waltham, MA, USA), superoxide dismutase (SOD) (Lot No. 706002, Cayman Chemical, Ann Arbor, MI, USA), and catalase (Lot No. 707002, Cayman Chemical, Ann Arbor, MI, USA) were measured as recommended by the manufacturer. The samples were diluted appropriately to lower the concentrations of serum enzymes to a range detectable by the respective ELISA kits.

The crude mucin in the samples was analyzed following the method described by Horn et al. [20] with some modifications. The collected jejunal digesta samples were first lyophilized for 96 h (Unitop 600 L, Virtis, Gardiner, NY, USA) and ground using a coffee grinder. Subsequently, 3 g of the lyophilized jejunal digesta was transferred to an open-ended, round-bottom 45-mL ultracentrifuge tube. Afterward, 20 mL of cold NaCl solution (0.15 mol/L NaCl, 0.02% NaN_3_, kept at 4 °C) was added to the sample, and the content was homogenized for 1 min (T25 Basic, IKA Corp., Staufen, Germany). The resulting mixture was centrifuged at 12,000 × *g* at 4 °C for 20 min, and the supernatant was carefully dispensed into a pre-weighed 50-mL plastic centrifuge tube (Greiner Bio-One, Monroe, NC, USA). Mucin proteins were extracted by adding 15 mL of cold (4 °C) absolute ethanol to the supernatant. The mixture was kept at −20 °C overnight. Afterward, the mixture was centrifuged at 1400 × *g* for 10 min at 4 °C, and the mucin pellet at the base of the tube was retained. The mucin pellet was washed with a mix of cold (4 °C) 15 mL absolute ethanol and 20 mL NaCl solution and kept overnight at −20 °C. After the mixture was retrieved from the freezer, it was centrifuged at 1400 × *g* for 10 min at 4 °C, and the resulting pellet was retained. The pellet was then rewashed twice with chilled absolute ethanol until the supernatant was clear. Subsequently, the samples were placed on ice in a fume hood to evaporate the leftover ethanol completely. Water was removed by suction from the mucin pellet, and the pellet was weighed to obtain the crude mucin yield.

### Chemical analysis

The experimental diets and dry excreta samples were ground using a centrifugal grinder (ZM 200; Retsch GmbH, Haan, Germany), and the dried ileal digesta samples were ground using a coffee grinder. The ground samples were then dried at 105 °C in a forced-air drying oven (Precision Scientific Co., Chicago, IL, USA; method 934.01 [21]) until a constant weight was observed for dry matter (DM) determination. Gross energy (GE) in the samples was analyzed using an isoperibol bomb calorimeter (Parr 6200; Parr Instrument Co., Moline, IL, USA), and nitrogen (N) using the combustion method (TruMac® N; LECO Corp., St. Joseph, MI, USA; method 990.03 [22]). The concentration of titanium was measured with a microplate reader at an absorbance of 410 nm following the technique outlined by Myers et al. [23].

### Calculations

Using the index method, the TTR and AID (%) of nutrients were calculated using the outlined equation [24]:

$$\textrm{TTR}\ \textrm{or}\ \textrm{AID},\%=100-\left[\left({\textrm{Ti}}_{\textrm{I}}/{\textrm{Ti}}_{\textrm{O}}\right)\times \left({\textrm{D}}_{\textrm{O}}/{\textrm{D}}_{\textrm{I}}\right)\times 100\right]$$

where Ti_I_ and Ti_O_ are the concentrations of titanium (g/kg DM) in diets, and excreta/ileal digesta samples, respectively; D_I_ and D_O_ are the concentration of nutrients (g/kg DM) in diets and excreta or ileal digesta samples, respectively.

The AID of energy and the apparent metabolizable energy (AME; kcal/kg DM) of the diet were computed as a product of the coefficient and GE concentrations (kcal/kg) in the diet. Using a factor of 8.22 kcal/g N, the nitrogen corrected AME (AMEn) was computed by correcting for zero N retention following the method outlined by Zhang and Adeola [25].

$$\textrm{AMEn}\ \left(\textrm{kcal}/\textrm{kg}\;\textrm{DM}\right )=\left[\textrm{AME}-\left(8.22\times \textrm{Nrt}\right)\right]$$

where Nrt = N retention in g/kg of DM intake. The Nrt was calculated as outlined below:

$$\textrm{Nrt}\ \left(\textrm{g}/\textrm{kg}\ \textrm{DM}\right)={\textrm{N}}_{\textrm{I}}-\left[{\textrm{N}}_{\textrm{O}}\times \left({\textrm{Ti}}_{\textrm{I}}/{\textrm{Ti}}_{\textrm{O}}\right)\right]$$

where N_I_ and N_O_ are the N concentrations (g/kg DM) in the diet and excreta, respectively.

### Statistical analysis

Pre-coccidia challenge growth performance and nutrient digestibility data were analyzed using the MIXED procedure of SAS (SAS Inst. Inc., Cary, NC, USA). The 3 diets were considered as fixed effects and the 16 blocks as random effects. Post-coccidia challenge data were analyzed using the MIXED procedure of SAS as a 3 × 2 factorial: three levels of ETY (0, 1, or 2 g/kg) with two coccidia challenge states (challenge or no-challenge) for main effects. The 6 treatments were considered as fixed effects, and 8 blocks as random effects. The initial body weight of birds was the blocking factor. Orthogonal polynomial contrasts were used to estimate the linear and quadratic effects of ETY. The growth performance data from d 14 to 21 were analyzed using the MIXED procedure as an ANCOVA to adjust for the difference in d 14 BW. Oocyst count and lesion scoring data were log-transformed and analyzed for the effect of ETY on challenged birds. There were no detectable oocysts or lesions in the non-challenged birds. Outliers, defined as values outside of ±1.5 × interquartile range, were identified and removed. Statistical significance and tendency were declared at *P* < 0.05 and 0.05 ≤ *P* < 0.10, respectively. Cage was considered as the experimental unit.

## Results

### Growth performance and nutrient digestibility

Pre-coccidia challenge growth performance and nutrient digestibility of broiler chickens fed increasing levels of dietary ETY from d 1 to 14 post hatching are shown in Table 3. There was a linear increase (*P* < 0.05) in G:F from d 1 to 14 as ETY supplementation increased in the diets of broiler chickens. There was no effect of ETY supplementation on the TTR of DM, GE, N, or the AME from d 1 to 14. The main effects of ETY and coccidia challenge on the growth performance and nutrient digestibility of broiler chickens from d 14 to 21 post hatching are summarized in Table 4. Due to the absence of significant interaction between ETY and coccidia challenge, main effects are presented. Six observations in the nutrient digestibility data for the challenge group were identified as outliers and designated as missing observations. Supplementation with ETY linearly increased (*P* < 0.05) the G:F of broiler chickens irrespective of the challenge state. Coccidia challenge (CC) decreased *(P* < 0.01) the BW of broiler chickens on d 21. The CC also decreased (*P* < 0.05) BW gain, FI and G:F. Dietary ETY linearly increased (*P* < 0.01) the AID of DM, N, and GE as well as the ileal digestible energy (IDE) in broiler chickens regardless of the challenge state. However, there was no ETY effect on the TTR of DM, N, the AME, or AMEn. Challenging birds with *Eimeria* spp. decreased (*P* < 0.01) the AID of DM, N, GE, and the IDE. The TTR of DM and GE in broiler chickens, as well as the AME and AMEn of diets also reduced (*P* < 0.01) with the CC.

**Table 3:** Growth performance and nutrient digestibility of broiler chickens fed ETY supplemented diets, d 1 to 14 post hatching^a^

Item	Concentrations of ETY, g/kg			SD^**b**^	***P***-value	
	0	1	2		L	Q
Growth performance						
BW, g						
d 1	49.9	49.9	49.9	5.89	–	–
d 7	144.5	145.1	146.1	13.85	0.440	0.902
d 14	367.4	362.1	375.0	34.56	0.377	0.220
d 1 to 7						
BW gain, g/bird	94.6	95.2	96.2	8.71	0.439	0.905
Feed intake, g/bird	136.2	133.4	136.4	17.78	0.966	0.382
G:F, g/kg	699.4	718.6	708.7	65.64	0.691	0.472
d 7 to 14						
BW gain, g/bird	223.0	217.0	228.9	23.94	0.409	0.155
Feed intake, g/bird	340.2	326.8	328.4	34.93	0.285	0.432
G:F, g/kg	657.7	663.5	699.0	43.01	0.006	0.231
d 1 to 14						
BW gain, g/bird	317.5	312.2	325.1	30.72	0.377	0.220
Feed intake, g/bird	476.4	460.2	464.7	47.08	0.331	0.319
G:F, g/kg	668.5	678.0	700.8	36.98	0.017	0.553
Nutrient digestibility						
TTR of DM, %	70.3	70.0	70.3	1.43	0.926	0.462
TTR of GE, %	72.9	73.0	73.2	1.66	0.721	0.894
TTR of N, %	57.8	57.1	58.5	2.41	0.388	0.167
AME, kcal/kg DM	2932.1	2934.1	2949.6	66.98	0.462	0.745

*ETY* Enzymatically treated yeast, *L* Linear effect of ETY, *Q* Quadratic effect of ETY, *BW* Body weight, *G:F* Gain to feed ratio, *TTR* Total tract retention, *DM* Dry matter, *GE* Gross energy, *N* Nitrogen, *AME* Apparent metabolizable energy

^a^Data are means of 16 replicate cages, 10 birds per cage were used from d 1 to 14 post hatching

^b^*SD* Standard deviation

**Table 4:** Main effects of ETY and coccidia challenge on the growth performance and nutrient digestibility of broiler chickens, d 14 to 21 post hatching

Item	Concentrations of ETY, g/kg			CS		SD^**b**^	***P***-value			
							Challenge	ETY		Challenge × ETY interaction
	0	1	2	No-challenge	Challenge			L	Q	
Growth performance^a^										
BW, g										
d 14	392.8	385.6	398.6	392.0	392.6	62.99	–	–	–	–
d 21	732.0	727.2	736.5	799.2	664.6	29.34	< 0.001	0.665	0.436	0.674
BW gain, g/bird	339.7	334.9	344.2	406.9	272.3	29.34	< 0.001	0.665	0.436	0.674
Feed intake, g/bird	524.7	467.4	482.5	523.3	459.8	69.27	0.012	0.095	0.099	0.549
G:F, g/kg	659.3	708.2	707.7	781.9	601.5	66.61	< 0.001	0.025	0.178	0.946
No. of replicates	16	16	16	24	24					
Nutrient digestibility										
AID of DM, %	55.6	58.0	61.3	65.6	50.9	4.99	< 0.001	0.003	0.694	0.572
AID of GE, %	55.2	57.8	62.2	68.1	48.6	5.52	< 0.001	0.001	0.428	0.346
AID of N, %	65.6	67.1	70.0	77.3	57.8	4.71	< 0.001	0.003	0.444	0.224
IDE, kcal/kg DM	2220.0	2323.0	2506.3	2742.2	1957.3	222.03	< 0.001	0.001	0.399	0.348
TTR of DM, %	63.0	62.3	63.1	73.8	51.8	2.44	< 0.001	0.943	0.271	0.693
TTR of GE, %	63.1	62.5	63.4	76.8	49.2	2.33	< 0.001	0.648	0.272	0.885
TTR of N, %	55.7	54.4	55.1	67.0	43.2	3.31	< 0.001	0.550	0.354	0.501
AME, kcal/kg DM	2535.4	2512.8	2557.5	3089.6	1980.9	93.88	< 0.001	0.486	0.223	0.874
AMEn, kcal/kg DM	2387.3	2371.4	2419.8	2913.2	1872.5	86.44	< 0.001	0.279	0.214	0.878
No. of replicates	15	14	13	24	18					

*ETY* Enzymatically treated yeast, *CS* Challenge state, *L* Linear effect of ETY, *Q* Quadratic effect of ETY, *BW* Body weight, *G:F* Gain to feed ratio, *AID* Apparent ileal digestibility, *IDE* Ileal digestible energy, *TTR* Total tract retention, *DM* Dry matter, *GE* Gross energy, *N* Nitrogen, *AME* Apparent metabolizable energy, *AMEn* Nitrogen corrected apparent metabolizable energy

^a^Analysis of covariance was used to adjust for the difference in BW on d 14

^b^*SD* Standard deviation

### Gene expression, serum antioxidants and mucin yield

The main effects of ETY and coccidia challenge on the relative expression of genes in the cecal mucosa, serum antioxidant markers and jejunal mucin yield in broiler chickens on d 21 are summarized in Table 5. One outlier was removed from the challenge group in the gene expression data. In the serum and mucin data, 4 observations from each of the no-challenge and challenge groups were removed as outliers and due to hemolysis of blood samples. There was a tendency (*P* = 0.08) for ETY to reduce *IL-1β* gene expression in broiler chickens. However, there was no main effect of ETY on the gene expression of *TNFα*, *IL-10*, and *IL-6*. Dietary supplementation of ETY linearly increased (*P* < 0.05) the relative gene expression of *OCLN* in the cecal mucosa of broiler chickens but not *CLDN1*. The CC increased (*P* < 0.05) the mRNA gene expression of *TNFα*, *IL-1β*, *IL-10*, and *IL-6*. Dietary supplementation with ETY linearly increased (*P* < 0.05) serum catalase in broiler chickens regardless of the challenge state. There were quadratic responses (*P* < 0.05) in the serum catalase and GPX activities in broiler chickens fed increasing levels of ETY in the diet on d 21. However, dietary ETY supplementation did not affect the serum SOD activity. The CC decreased (*P* < 0.05) serum catalase and increased (*P* < 0.01) the jejunal mucin yield in broiler chickens, but there was no ETY effect.

**Table 5:** Main effects of ETY and coccidia challenge on the relative expression of genes in the cecal mucosa, serum antioxidant markers and jejunal mucin yield in broiler chickens, d 21 post hatching

Item	Concentrations of ETY, g/kg			CS		SD^**a**^	***P***-value			
							Challenge	ETY		Challenge × ETY interaction
	0	1	2	No-challenge	Challenge			L	Q	
Relative gene expression										
*TNFα*	0.91	0.93	0.85	0.75	1.04	0.188	< 0.001	0.385	0.361	0.297
*CLDN1*	0.85	0.89	0.88	0.85	0.90	0.496	0.739	0.919	0.824	0.251
*IL-1β*	0.90	0.77	0.68	0.61	0.95	0.364	0.002	0.084	0.855	0.979
*OCLN*	0.68	1.10	1.15	0.97	0.98	0.797	0.919	0.045	0.333	0.535
*IL-10*	0.59	0.82	0.70	0.32	1.09	0.730	0.001	0.705	0.410	0.491
*IL-6*	0.91	0.58	0.55	0.33	1.03	0.877	0.005	0.220	0.546	0.733
No. of replicates	16	16	15	24	23					
Serum antioxidants										
Catalase, U/mL	2.88	9.95	7.88	8.85	4.96	6.744	0.049	0.044	0.029	0.265
SOD, U/mL	629.27	574.94	529.44	661.03	494.73	366.514	0.075	0.314	0.977	0.095
GPX, U/mL	12.12	12.35	12.15	12.22	12.19	0.199	0.505	0.589	0.002	0.920
Mucin, mg/g jejunal digesta	383.63	348.96	362.93	309.36	420.99	77.002	< 0.001	0.438	0.295	0.627
No. of replicates	13	13	14	20	20					

*ETY* Enzymatically treated yeast, *CS* Challenge state, *L* Linear effect of ETY, *Q* Quadratic effect of ETY, *TNFα* Tumor Necrosis factor α, *CLDN1* Claudin 1, *IL* Interleukin, *OCLN* Occludin, *SOD* Superoxide dismutase, *GPX* Glutathione peroxidase

^a^*SD* Standard deviation

### Intestinal morphology

**Fig. 2:**
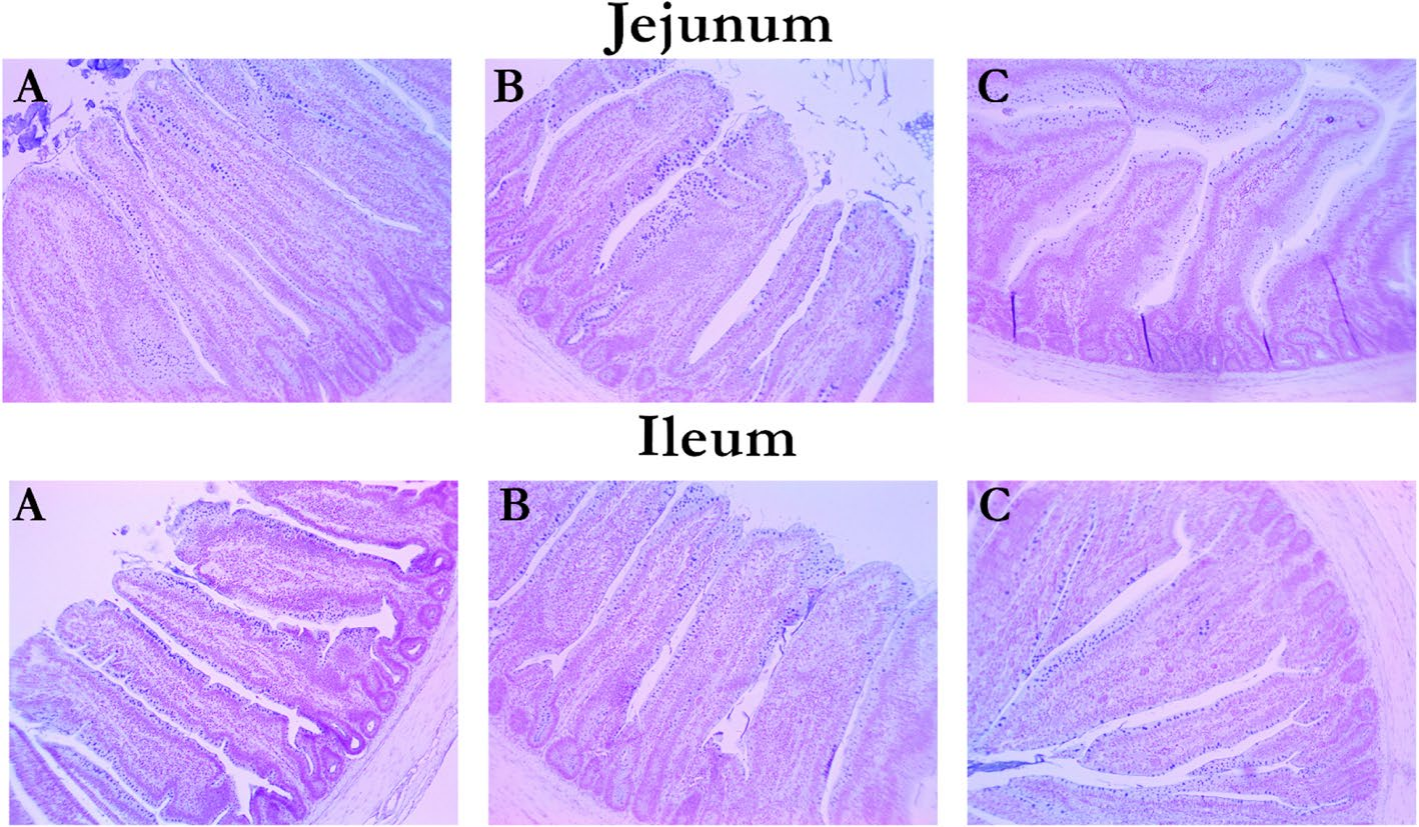
Effects of dietary enzymatically treated yeast and coccidia challenge on the jejunal and ileal morphology of broiler chickens on d 21. Representative images showing the jejunal and ileal segments of broiler chickens stained with Alcian blue and periodic acid-Shiff (AB-PAS). **A** Birds fed 0 g/kg ETY diet; (**B**) Birds fed 1 g/kg ETY diet; (**C**) Birds fed 2 g/kg ETY diet. Magnification: × 10

The main effects of ETY and coccidia challenge on the intestinal morphology, the goblet cell count and density of broiler chickens fed ETY-supplemented diets on d 21 are summarized in Table 6. Images of jejunal and ileal tissues of broiler chickens at d 21 stained with AB-PAS are shown in Fig. 2. One outlier was removed from the challenge group in the intestinal morphology data. Supplementation with dietary ETY linearly increased (*P* < 0.05) the ileal VH:CD ratio, GC count, and density in broiler chickens regardless of the challenge state. Coccidia challenge increased (*P* < 0.01) ileal and jejunal crypt depths in broiler chickens on d 21. Moreover, the CC decreased (*P* < 0.01) ileal and jejunal VH:CD ratios and the jejunal villus height in broiler chickens.

**Table 6:** Main effects of ETY and coccidia challenge on the intestinal morphology and goblet cells of broiler chickens, d 21 post hatching^a^

Item	Concentrations of ETY, g/kg			CS		SD^**a**^	***P***-value			
							Challenge	ETY		Challenge × ETY interaction
	0	1	2	No-challenge	Challenge			L	Q	
Ileum										
Villi height, μm	529.9	564.9	608.3	539.4	596.0	143.53	0.180	0.131	0.930	0.848
Crypt depth, μm	208.0	157.4	196.2	144.9	229.5	52.55	< 0.001	0.536	0.009	0.238
VH:CD ratio	2.9	3.6	3.5	3.8	2.8	0.76	< 0.001	0.042	0.083	0.677
GC count (cells/villi)	90.1	116.9	122.5	112.1	107.5	24.75	0.478	< 0.001	0.142	0.972
GC density (cells/μm of VH)	0.8	1.0	1.1	1.0	0.9	0.27	0.101	0.030	0.255	0.540
Jejunum										
Villi height, μm	643.6	640.3	640.7	710.1	573.0	111.19	< 0.001	0.974	0.954	0.583
Crypt depth, μm	266.0	275.7	285.6	180.3	371.2	66.01	< 0.001	0.400	0.984	0.919
VH:CD ratio	3.0	3.0	2.8	4.2	1.7	0.76	< 0.001	0.476	0.633	0.451
GC count (cells/villi)	75.4	79.3	78.9	73.2	82.6	22.82	0.194	0.576	0.731	0.446
GC density (cells/μm of VH)	0.6	0.6	0.6	0.5	0.7	0.20	0.012	0.771	0.859	0.986
No. of replicates	16	15	16	24	23					

*ETY* Enzymatically treated yeast, *CS* Challenge state, *L* Linear effect of ETY, *Q* Quadratic effect of ETY, *VH:CD ratio* Villus height to crypt depth ratio, *GC* Goblet cell, *VH* Villus height

^a^*SD* Standard deviation

### Lesion scores and oocyst count

The log-transformed ileal, and intestinal lesion scoring in coccidia-challenged broiler chickens fed ETY-supplemented diets at d 21 are shown in Table 7. Lesions were scored and oocysts were counted only in birds challenged with *Eimeria* spp., as they were not detectable in unchallenged birds. The oocysts in the excreta and ileal digesta of coccidia-challenged broiler chickens showed a quadratic response (*P* < 0.05) with increasing supplementation of ETY in the diets on d 21. However, supplementation with dietary ETY did not affect the *Eimeria* lesion scores in coccidia-challenged broiler chickens.

**Table 7:** Log-transformed oocyst count and intestinal lesion scoring in coccidia-challenged broiler chickens fed ETY supplemented diets, at d 21 post hatching^a^

Item	Concentrations of ETY, g/kg			SD^**b**^	***P***-value	
	0	1	2		L	Q
Oocyst count						
Oocysts in excreta	5.4	5.2	5.2	0.17	0.006	0.014
Oocysts in ileal digesta	5.6	5.3	5.2	0.17	< 0.001	0.026
Lesion scoring						
Lesion score *E. tenella*	2.6	2.0	2.1	1.10	0.213	0.279
Lesion score *E. acervulina*	1.3	0.6	0.9	1.24	0.404	0.264
Lesion score *E. maxima*	2.3	1.4	1.6	1.49	0.253	0.236

*ETY* Enzymatically treated yeast, *L* Linear effect of ETY, *Q* Quadratic effect of ETY, *E. Eimeria*

^a^Data are means of 8 replicate cages

^b^*SD* Standard deviation

## Discussion

The current study investigated the effects of dietary supplementation of enzymatically treated yeast in broiler chickens subjected to a coccidia challenge. The challenge model used in the current study caused a moderate form of coccidiosis as only two birds died during the post-challenge period. The G:F was significantly improved by dietary supplementation of ETY in broiler chickens before the coccidia challenge. Dietary yeast cell wall components have been reported to stimulate growth performance and improve feed efficiency in broiler chickens by promoting intestinal development [7, 26].

Several studies have shown that the most pronounced effects of coccidiosis in broiler chickens are growth retardation, reduced nutrient utilization, and diminished growth efficiency [15, 27]. In the current study, the CC reduced BW gain, FI, and G:F in broiler chickens. Conversely, dietary supplementation of ETY improved the G:F of broiler chickens regardless of the challenge state. This improvement is likely due to the improved ileal digestibility of nutrients observed, specifically energy, N and DM digestibility. However, ETY did not completely mitigate the effect of the CC on growth performance.

The CC reduced the AID of DM, N, and energy, which corroborates studies that reported a CC-induced decline in nutrient utilization in broiler chickens [27, 28]. The reduced nutrient utilization due to the CC may be responsible for the reduction in growth performance observed in coccidia-challenged birds [27]. Dietary ETY could be efficacious in stimulating nutrient utilization in broiler chickens regardless of their challenge state; we observed improvement in the ileal digestibility of DM, N, and energy in broiler chickens fed ETY diets. The IDE also increased with higher levels of ETY in the diet. These results are linked to the ability of ETY to promote a healthy intestinal environment [10, 29]. In the same vein, yeast cell wall products have been reported to significantly improve pancreatic activity and the release of trypsin in chickens, which may be responsible for the improved AID of N observed in this experiment [30]. However, the absence of an ETY effect on the TTR of N in broiler chickens may be due to the hindgut protein fermentation, which may confound excreta N digestibility coefficients [31, 32].

Cytokines are essential for basic processes in the body, including immunity, inflammation, and metabolism. Their function in inflammation qualifies them as a gauge for measuring cellular immunity during active infection [33]. A cascade of events after a pathogenic invasion can increase *IL-6*, *TNFα*, *IL-10*, and *IL-1β* levels, as seen in the current study [34, 35]. A tendency for dietary ETY to reduce cecal *IL-1β* expression and increase *OCLN* expression may be attributed to the anti-inflammatory and barrier-protecting properties of yeast β-glucans and mannans [36, 37]. However, ETY did not affect *CLDN1* expression. The coccidia parasite substantially damages the intestinal tract of birds by producing sporozoites that infiltrate the intestinal walls [38]. Pathogenic interference of the intestinal tight junctions can increase gut permeability and reduce nutrient absorption due to loss of cell polarity [39]. In the current study, the 6-day CC did not affect the gene expression of *OCLN* and *CLDN1* in broiler chickens. However, several studies have reported contrasting results. Hansen et al. [40] saw a CC-induced reduction in *OCLN* at 7 d post infection and a CC-induced reduction in junctional adhesion molecule 3 (*JAM3*) at 10 d post infection. On the other hand, Teng et al. [41] reported a CC-induced upregulation in *CLDN1* and *JAM2* at 6 d post infection. Hence, there is a need for further research on the effect of coccidiosis and the duration of coccidia infection on tight junction proteins.

The production of antioxidant enzymes such as catalase and SOD in the blood is needed to remove excess reactive oxygen species (ROS) in the body [42]. In the current study, ETY maintained the oxidative status of broiler chickens, which relates to the increased serum catalase regardless of the challenge state. This increase is attributable to the β-glucan and mannan components of ETY, which have been reported to actively scavenge ROS and modulate cytokine-induced cytotoxicity [43–45] and is in concordance with the improved feed efficiency observed. Moreover, an increased antioxidation status in chickens is positively correlated with improved growth performance [46]. On another note, the CC reduced serum catalase, evidently demonstrating a possible CC-induced reduction in the antioxidation capacity of broiler chickens. The negative effects of a mixed *Eimeria* infection on serum antioxidants have also been reported [47, 48]. Abdelhady et al. [49] reported a coccidia-induced reduction in the serum SOD and glutathione peroxidase in broiler chickens.

The intestinal morphology and health of animals can be assessed by the villus height, crypt depth, and VH:CD ratio [50, 51]. An increase in ileal VH:CD ratio in ETY-fed broiler chickens indicates a lower epithelial turnover rate, which is suggestive of a contributory role in a healthy gut. On the other hand, the deeper crypts and reduced VH:CD ratio in the intestine of birds due to the CC may lead to inefficient nutrient uptake due to a faster tissue turnover rate [51, 52]. The exact approach by which ETY increased intestinal GC count and density in broiler chickens is unclear. However, this increase may be related to the immune-modulatory actions of the mannan component of ETY [53, 54]. Mucins prevent the entry of antigens into the blood, and they contain active IgA, which initiates pathogen binding [55]. During an infection such as coccidiosis, intestinal mucin secretion may be upregulated due to the exocytosis of mucin granules from goblet cells [56]. It is noteworthy that increased jejunal mucin yield corresponds to the CC-induced increase in jejunal mucin-secreting goblet cell density in the current study. Further studies are needed to investigate the effect of ETY on the intestinal GC count and density in coccidia-challenged broiler chickens.

To our knowledge, there are no data on the effect of yeast cell extracts on ileal digesta oocyst count in broiler chickens subjected to a CC. Nevertheless, there is a possibility that yeast-derived mannans can activate C-type lectins, which interact with surface polysaccharides of different parasites, thereby inhibiting parasitic maturation [57–60]. Hence, an observed reduction in oocysts in the ileal digesta and excreta of coccidia-challenged birds indicates an ETY-derived opposition to oocyst multiplication. Further studies may be needed to evaluate the mechanism of an ETY-induced reduction in oocyst count and how that may influence intestinal health and development.

## Conclusions

Our results showed that dietary enzymatically treated yeast significantly improved the feed efficiency, nutrient digestibility, antioxidation status, and intestinal morphology of broiler chickens regardless of their challenge state. Hence, dietary enzymatically treated yeast at 2 g/kg may be favorable for growth promotion and maintaining intestinal development in broiler chickens. However, dietary ETY supplementation did not completely alleviate the adverse effects of a coccidia challenge in broiler chickens. Therefore, further studies are required in *Eimeria*-challenged broiler chickens fed diets supplemented with enzymatically treated yeast.

## Supplementary Information

**Additional file 1: Table S1.** Chemical composition of enzymatically treated yeast.

## Data Availability

All data from this study are available from the corresponding author upon reasonable request.

## Abbreviations

AB-PAS: Alcian blue and periodic acid-Schiff
AID: Apparent ileal digestibility
AME: Apparent metabolizable energy
BW: Body weight
CC: Coccidia challenge
DM: Dry matter
ETY: Enzymatically treated yeast
FI: Feed intake
G:F: Gain to feed ratio
GAPDH: Glyceraldehyde-3-phosphate dehydrogenase
GC: Goblet cells
GE: Gross energy
IDE: Ileal digestible energy
IL-1β: Interleukin 1β
IL-6: Interleukin 6
IL-10: Interleukin 10
JAM: Junctional adhesion molecule
ROS: Reactive oxygen species
TNFα: Tumor necrosis factor alpha
TTR: Total tract retention
VH:CD: Villus height to crypt depth
