# Supplementary material for: Growth performance, nutrient digestibility, intestinal morphology, cecal mucosal cytokines and serum antioxidant responses of broiler chickens to dietary enzymatically treated yeast and coccidia challenge
Source: J Anim Sci Biotechnol. 2023 Apr 10;14:57. doi: 10.1186/s40104-023-00846-z (PMC10084602; doi:10.1186/s40104-023-00846-z)
Supplement: Supplementary file 1 — Additional file 1: Table S1. Chemical composition of enzymatically treated yeast. [file 40104_2023_846_MOESM1_ESM.docx]

**Growth performance, nutrient digestibility, intestinal morphology, cecal mucosal cytokines and serum antioxidant responses of broiler chickens to dietary enzymatically treated yeast and coccidia challenge**

Emmanuel O. Alagbe, Hagen Schulze, and Olayiwola Adeola

**Additional file 1**

**Table S1** Chemical composition of enzymatically treated yeast^1^

| **Item** | **Value** |
| --- | --- |
| Gross energy, kcal/kg | 4441.28 |
| Dry matter, % | 92.00 |
| Crude Protein, % | 36.00 |
| Crude Fiber, % | 2.00 |
| Ash, % | 12.00 |
| Ether extract, % | 1.00 |
| Glucan, % | 28.00 |
| Mannan, % | 12.00 |
| Amino acids (% CP) |  |
| Indispensable amino acids |  |
| Arginine | 3.88 |
| Histidine | 2.07 |
| Isoleucine | 4.86 |
| Leucine | 6.78 |
| Lysine | 7.30 |
| Methionine | 1.61 |
| Phenylalanine | 4.05 |
| Threonine | 5.11 |
| Valine | 5.67 |
| Dispensable amino acids |  |
| Alanine | 6.22 |
| Aspartic acid | 9.78 |
| Cysteine | 0.65 |
| Glutamic acid | 10.29 |
| Glycine | 4.20 |
| Proline | 3.34 |
| Serine | 5.35 |
| Tyrosine | 2.93 |

^1^The enzyme used in yeast hydrolyzation is generally recognized as safe (GRAS) and it meets the specifications laid down by the Joint FAO/WHO Expert Committee on Food Additives and the Food Chemicals Codex
